# Target deconvolution of a selective HepG2 cytotoxic hit identifies GSTP1 and TRAP1 as candidate molecular targets

**DOI:** 10.1039/d6ra04601k

**Published:** 2026-07-10

**Authors:** Belal O. Al-Najjar, M. Helal, Fadi G. Saqallah, B. Bandy

**Affiliations:** a Department of Pharmaceutical Sciences, Faculty of Pharmacy, Al-Ahliyya Amman University Amman 19328 Jordan b.najjar@ammanu.edu.jo; b Physiology, Pharmacology, and Toxicology Division, Biomedical Sciences Department, Faculty of Medicine and Health Sciences, An-Najah National University Nablus Palestine; c Faculty of Pharmacy, Al-Zaytoonah University of Jordan Amman 11733 Jordan; d College of Pharmacy and Nutrition, University of Saskatchewan Saskatoon SK S7N 5E4 Canada

## Abstract

Hepatocellular carcinoma (HCC) remains a lethal malignancy with limited therapeutic options in advanced disease, highlighting the need to identify selective cytotoxic agents and biologically relevant molecular targets for early anticancer drug discovery. In this study, a focused in-house compound set was screened against HepG2, MCF-7, and MDA-MB-231 cancer cell lines using the MTT assay to identify compounds with selective activity toward HCC-derived cells. The most active hit, HTS00019, was then subjected to an integrated computational target-deconvolution workflow comprising SEA target prediction, reverse docking against cancer-relevant proteins, binding-mode analysis, 100 ns molecular dynamics simulations, MM-GBSA binding free energy estimation, and *in silico* toxicological profiling using ProTox-3.0. HTS00019 showed potent and selective cytotoxicity toward HepG2 cells, with an IC_50_ value of 1.70 ± 0.22 µM and approximately 50-fold selectivity over MCF-7 and MDA-MB-231 cells. SEA and reverse docking analyses prioritized GST-family proteins and TRAP1 as likely candidate targets. Docking suggested favorable binding within the GSTP1 H-site and the ATP-associated region of TRAP1, while molecular dynamics simulations supported stable accommodation of the compound in both proteins, with stronger ligand positional stability in TRAP1. MM-GBSA analysis further favored TRAP1 binding, with a calculated Δ*G*_bind_ of −75.49 kcal mol^−1^ compared with −59.98 kcal mol^−1^ for GSTP1. Interaction analysis indicated hydrophobic and ionic contacts in GSTP1, whereas halogen bonding, π-cation, and hydrophobic interactions contributed to the predicted TRAP1 binding mode. *In silico* toxicological profiling classified HTS00019 as oral toxicity class 4 and flagged potential hepatotoxicity, mutagenicity, immunotoxicity, and carcinogenicity liabilities, while p53- and ATAD5-related stress pathway predictions were inactive. Overall, HTS00019 was identified as a selective HepG2 cytotoxic hit, with GSTP1 and TRAP1 emerging as computationally prioritized candidate targets. However, the absence of biochemical target validation and the predicted toxicological liabilities indicate that HTS00019 remains an early-stage hit requiring mechanistic confirmation and structural optimization.

## Abstract 2

A selective HepG2 cytotoxic hit was identified and computationally linked to GSTP1 and TRAP1 through SEA, docking, MD, and MM-GBSA analyses. Toxicity alerts indicate an early hit requiring validation and optimization.

## Graphical abstract

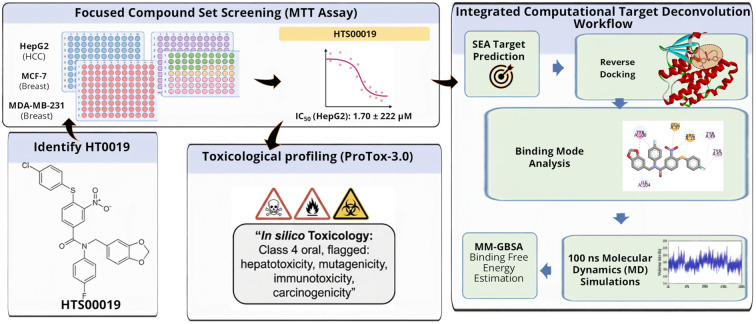

## 1. Introduction

Hepatocellular carcinoma (HCC) is the sixth most frequent type of malignancy and the third leading cause of cancer deaths worldwide, accounting for 906 000 new cases and 830 000 deaths every year, as per the latest statistics on cancer incidence worldwide.^1^ The current trend of HCC incidence is a cause for concern, with rising numbers of cases in both developed and developing countries, primarily due to the growing incidence of metabolic syndrome, non-alcoholic fatty liver disease, and viral hepatitis infections. Notwithstanding the remarkable developments that have been made regarding HCC treatments during the last 20 years, the five-year survival rate for patients suffering from this condition still remains alarmingly low, being only under 20%.^2^

The management of HCC has changed significantly since the landmark SHARP study showed the benefits of sorafenib when used as the primary systemic treatment for patients with HCC in an advanced stage. Several tyrosine kinase inhibitors, including lenvatinib, regorafenib, and cabozantinib, have been approved over the past few years. Also, immune checkpoint inhibitors like atezolizumab and bevacizumab have entered the scene.^3^ Nevertheless, none of the treatment breakthroughs mentioned above have led to significant improvements in overall survival rates because of the biological diversity of HCC, the presence of acquired resistance mechanisms, and the lack of therapeutic windows in patients with compromised liver function. The biological intricacies associated with hepatocarcinogenesis, which are ex emplified by aberrant signal transduction pathways and an immune-suppressed tumor microenvironment, warrant the discovery of new molecular targets and the creation of novel treatment strategies.^4^

The glutathione S-transferase P1 (GSTP1) enzyme has drawn much interest as a drug target for hepatocellular carcinoma because it is over-expressed in the liver tumors, and it plays multiple roles in HCC pathogenesis. GSTP1 is a member of the glutathione S-transferase superfamily and catalyzes the conjugation of glutathione to electrophilic compounds, thus playing a role in the cellular detoxification process. In addition to its catalytic activity, GSTP1 has been shown to play a role in the regulation of apoptosis by interacting with key signaling molecules, such as c-Jun N-terminal kinase (JNK) and inhibitor of κB kinase (IKK). Recent meta-analyses have confirmed that GSTP1 hypermethylation and overexpression are significantly associated with the presence of HCC and poor prognosis, making this enzyme both a potential biomarker and a promising target for therapy.^5^

TNF receptor-associated protein 1 (TRAP1), a mitochondrial homolog of the heat shock protein 90 (HSP90) family, has also been identified as a promising target for cancer therapy. TRAP1 is often overexpressed in different types of cancers, including HCC, and is involved in the metabolic shift that characterizes cancer cells, triggering the Warburg effect due to the preference for aerobic glycolysis. According to the review article that was published in the *Journal Molecular Biotechnology*, the importance of HSP90 and TRAP1 in HCC treatment cannot be underestimated because these factors influence cancer cells' survival, ability to resist apoptosis, and metastasis potential.^6^

Combining phenotypic screening with target fishing *via* computation is a very promising strategy in modern drug discovery, which allows one to find bioactive compounds without knowing the targets involved. The special value of phenotypic screening is that it helps to discover compounds acting by physiological pathways, including multi-target and unknown pathways. The further use of target fishing techniques, such as SEA and reverse docking, makes it possible to quickly establish the targets of action, thus providing for a deeper understanding of mechanisms required for hit validation and optimization.^7,8^ Recent developments in the field of artificial intelligence have further improved the accuracy of target prediction, as illustrated in the 2025 study that successfully combined phenotypic screening with AI-driven target fishing for hit discovery efforts.^9^

This study demonstrates a proof-of-concept workflow integrating phenotypic screening with advanced computational tools to rapidly identify molecular origins of selective toxicity. This approach helped to characterize new anti-HCC compounds from our in-house compound library in previous research projects on various therapeutic areas, including anti-diabetic, anti-platelet, and anticancer hit discovery.^10–13^ The Maybridge Screening Collection, from which our library was derived, has been a rich source of valuable leads for drug discovery projects on various therapeutic areas. Our research efforts resulted in the identification of HTS00019 as a highly potent and highly selective cytotoxic compound against HepG2 hepatocellular carcinoma cells. By using the systematic approach of SEA target prediction and reverse docking analysis, we were able to identify GSTP1 and TRAP1 as the major molecular targets responsible for the observed activity, which served as a rational basis for the subsequent optimization of the structure–activity relationship of this promising lead compound.

## 2. Materials and methods

### 2.1 Chemicals and reagents

**Fig. 1:**
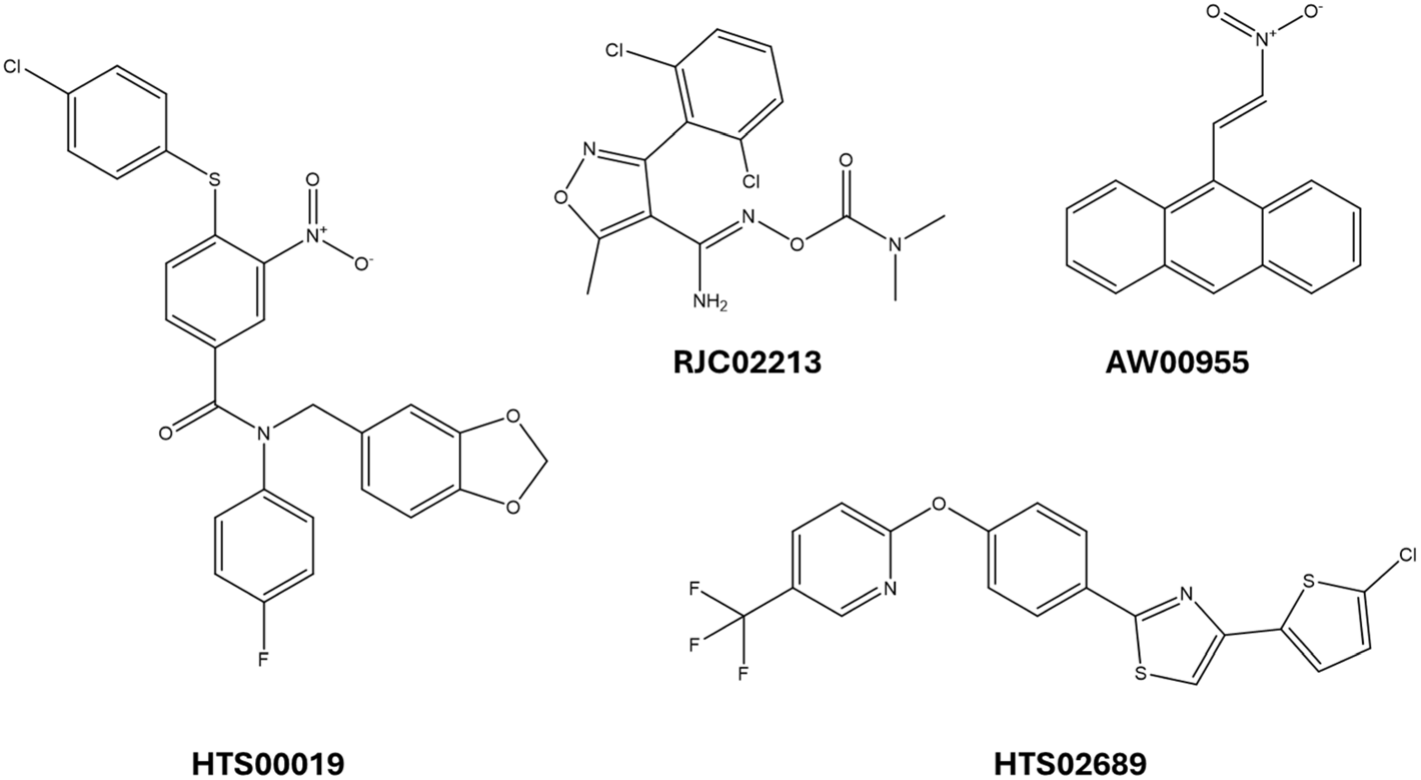
Chemical structures of the tested compounds. Two-dimensional chemical structures of compounds HTS00019, HTS02689, AW00955, and RJC02213. Structures were generated using ChemDraw Professional (version 16.0, PerkinElmer, Waltham, MA, USA).

All compounds tested in this study were sourced from an in-house chemical library at the Pharmacological & Diagnostic Research Centre, Al-Ahliyya Amman University. The compounds (Fig. 1) were purchased from the Maybridge Screening Collection (purity >95% by LC-MS/NMR). Given their commercial origin for early-stage screening, structural characterization and purity were verified by the supplier (Thermo Fisher Scientific, UK; Catalog No. HTS00019, HTS02689, AW00955, and RJC02213) consistent with their application in our previous investigations.^10–13^ Dulbecco's Modified Eagle Medium (DMEM), fetal bovine serum (FBS), l-glutamine, penicillin–streptomycin solution, and phosphate-buffered saline (PBS) were obtained from Gibco (Thermo Fisher Scientific, Waltham, MA, USA). The MTT (3-(4,5-dimethylthiazol-2-yl)-2,5-diphenyltetrazolium bromide) reagent was purchased from Sigma-Aldrich (St. Louis, MO, USA). All other chemicals and solvents used in this study were of analytical grade or higher purity.

### 2.2 Cell culture

Three human cancer cell lines were used in this study: MCF-7 (breast adenocarcinoma, ER-positive), MDA-MB-231 (breast adenocarcinoma, triple-negative), and HepG2 (hepatocellular carcinoma). These cell lines were purchased from the American Type Culture Collection (ATCC, Manassas, VA, USA) and cultured in DMEM media supplemented with 10% (v/v) FBS, 2 mM l-glutamine, 100 U per mL penicillin, and 100 µg per mL streptomycin. Cells were incubated at 37 °C in a humidified atmosphere with 5% CO_2_ and passaged every time they reached 80–90% confluence. Cell viability and morphology were checked periodically, and cells were used for experiments only if they were within 10 passages of thawing. Mycoplasma contamination was checked monthly using a PCR-based detection kit (Sigma-Aldrich) and found to be negative.

### 2.3 Cell viability assay

The cytotoxic potential of the test compounds was determined using the MTT colorimetric assay, which measures the activity of mitochondrial dehydrogenases in viable cells. In brief, cells in the logarithmic phase of growth were seeded at 5000 cells per well in 100 µL of medium in 96-well plates. Incubation overnight allowed for cell adherence, followed by replacing the culture medium with fresh medium supplemented with the experimental substances in concentrations from 0.01 to 100 µM using an eight-point concentration–response assay design. The control wells contained either the vehicle substance only (0.1% DMSO) or media lacking cells to serve as a negative background for absorbance measurement. All concentrations were run in triplicates and repeated independently three times.

After incubation for 48 hours with the compounds, 20 µL of the MTT solution (5 mg mL^−1^ in PBS) was added into each well, followed by further incubation for another 4 hours at 37 °C. The crystals formed after incubation were dissolved with 100 µL of DMSO in each well, and absorbance was then read at 570 nm on a microplate reader (BioTek Synergy H1, Winooski, VT, USA). Cell viability is reported as a percentage relative to untreated controls with the help of the following formula:Cell viability (%) = [(Abs_(sample)_ − Abs_(blank)_)/(Abs_(control)_ − Abs_(blank)_)] × 100

Concentration–response curves were generated, and half-maximal inhibitory concentration (IC_50_) values were determined by non-linear regression analysis using GraphPad Prism software (version 9.0, GraphPad Software, San Diego, CA, USA).

### 2.4 Target prediction using SEA search server

To generate suggestions on possible molecular targets for the active compounds identified in the cytotoxicity assay, we employed the Similarity Ensemble Approach (SEA) search server (https://sea.bkslab.org). This tool establishes correlations between sets of proteins by comparing the chemical similarity of their associated ligands.^7,8^

For the top hit compound, the two-dimensional structure was drawn using ChemDraw Professional (version 16.0, PerkinElmer, Waltham, MA, USA) and exported as a SMILES string. The SMILES entry was submitted to the SEA Search Server with default parameters, including the ECFP4 fingerprint type and a maximum *E*-value threshold of 10. The server generated a ranked list of potential protein targets based on statistical significance (*E*-value) and the Tanimoto coefficient of chemical similarity. Targets with *E*-values less than 1.0 × 10^−5^ were considered statistically significant and selected for further investigation.

### 2.5 Protein structure preparation

Three-dimensional structures of the selected protein targets were retrieved from the RCSB Protein Data Bank (PDB, https://www.rcsb.org).^14^ The following structures were used for molecular docking studies: GSTP1 (PDB: 10GS, resolution 2.20 Å), GSTM2 (PDB: 3GUR, resolution 2.50 Å), GSTA1 (PDB: 7BIB, resolution 2.03 Å), COPS5 (PDB: 5M5Q, resolution 2.20 Å), PDE7A (PDB: 4Y2B, resolution 2.20 Å), USP47 (PDB: 8ITP, resolution 3.00 Å), and TRAP1 (PDB: 7C7B, resolution 1.50 Å). These structures were selected based on the availability of co-crystallized ligands for binding mode validation.

Protein structures were prepared using AutoDockTools (version 1.5.7, The Scripps Research Institute, La Jolla, CA, USA). All water molecules were removed, polar hydrogen atoms were added, and Gasteiger partial charges were assigned to all atoms. The binding sites aimed for docking were defined based on the co-crystallized ligands and previous structural studies.

### 2.6 Ligand preparation

The two-dimensional structures of test compounds were converted to three-dimensional coordinates using Open Babel (version 3.1.1).^15^ All rotatable bonds were treated as flexible, allowing the ligand to adopt optimal conformations during docking. Partial charges were assigned using the Gasteiger–Marsili method, and the prepared ligand files were saved in PDBQT format to be used in AutoDock Vina.

### 2.7 Reverse docking studies

Reverse docking calculations were performed using AutoDock Vina, version 1.2.0, an established and validated tool for the process of molecular docking. For each target protein, the conformation space was defined as a 3D box surrounding the corresponding co-crystallized structure. In every pair of ligand and target, docking was performed such that nine docked poses were produced using an exhaustiveness value of 32. For every pose obtained, its binding energy was expressed as the predicted binding free energy in kcal mol^−1^. A greater negative value implies a tighter bond between the ligand and the protein. For every pair of ligand and target, the best binding pose was identified as that which had the lowest Δ*G* value. In order to prove the accuracy of our docking procedure, the co-crystalized ligands were extracted from their proteins and then docked back to their corresponding binding sites. The RMSD values calculated ranged less than 2.0 Å.^16^

### 2.8 Binding mode analysis

The binding modes of the best lead candidates were studied in depth using Discovery Studio Visualizer software (Version 2021, BIOVIA, San Diego, CA, USA). The protein–ligand interaction fingerprints were used to find important hydrogen bonding sites, hydrophobic contacts, and π–π interactions. The binding site was studied based on the amino acid residues found in a 4 Å region from the ligand, and the interaction energy was analyzed to understand the mode of selectivity.

### 2.9 Molecular dynamics simulation

All-atom molecular dynamics simulations were performed with the Desmond module implemented in the Schrödinger Maestro 2025-3 environment.^17^ Four independent systems were simulated, including the initial configurations consisted of the docked ligand–protein complexes of HTS00019 in complex with GSTP1 (PDB: 10GS), and in complex with TRAP1 (PDB: 7C7B), as well as the respective co-crystallized ligands TER117 (GSTP1) and SJT009 (TRAP1) in their native protein complexes. The complexes were embedded in an orthorhombic simulation box with a 10 Å solvent buffer. The systems were solvated using the TIP4P water model,^18^ and an ionic strength of 0.15 M NaCl was introduced to approximate physiological conditions. Long-range electrostatic interactions were treated using the particle mesh Ewald method, while van der Waals interactions were truncated at a cutoff distance of 9.0 Å.^19^

Following system preparation, energy minimization and equilibration were carried out according to the standard Desmond relaxation workflow, involving both *NVT* and *NPT* ensembles. Production simulations were subsequently conducted for 100 ns under *NPT* conditions with periodic boundary conditions, employing the OPLS4 force field.^20^ Temperature and pressure were maintained at 310 K and 1 atm, respectively, using the Nosé–Hoover chain thermostat and the Martyna–Tobias–Klein barostat.

The equilibration phase followed a multistage protocol beginning with Brownian dynamics at 10 K, followed by a sequence of restrained and unrestrained simulations in both *NVT* and *NPT* ensembles. This stepwise approach ensured gradual system relaxation prior to the final unrestrained 100 ns production run conducted under constant temperature and pressure conditions.

## 3. Results and discussion

### 3.1 Phenotypic screening identifies HTS00019 as a potent and selective HepG2 cytotoxic agent

A phenotypic screen of three different human cancer cell lines (MCF-7, MDA-MB-231, and HepG2) was carried out to test the cytotoxic effects of various drugs. The data revealed that the cytotoxic effects showed considerable variations. It has been observed that HTS00019 is very potent against the HepG2 cell line (a model of hepatocellular carcinoma), which had an IC_50_ value of 1.7 ± 0.22 µM. The potency of HTS00019 (IC_50_ = 1. 7 ± 0.22 µM) significantly exceeds that of FDA-approved HCC standards, such as Sorafenib (IC_50_ = 7.35–10.9 µM) in the HepG2 model.^21^ The potency of compound HTS00019 is particularly noteworthy in comparison to the other tested compounds: compound AW00955 showed moderate cytotoxicity with an IC_50_ value of 2.1 ± 0.05 µM against the HepG2 cell line, while compound RJC02213 showed low potency with an IC_50_ value of 8 ± 1.45 µM, and compound HTS02689 was found to be inactive with an IC_50_ value > 100 µM.

One of the most remarkable finds of this screening project is the highly selective profile of HTS00019. This compound has demonstrated IC_50_ values of 86.29 ± 1.50 µM for the MDA-MB-231 cell line and 94.03 ± 1.80 µM for the MCF-7 cell line. It has achieved selectivity indices of 50-fold and 55-fold for the HepG2 cell line compared to the breast cancer cell lines. It is highly likely that the compound interacts with a molecular target or pathway that is either uniquely crucial for the growth of hepatocellular carcinoma cells or is expressed differently compared to the other tested cell lines.

**Fig. 2:**
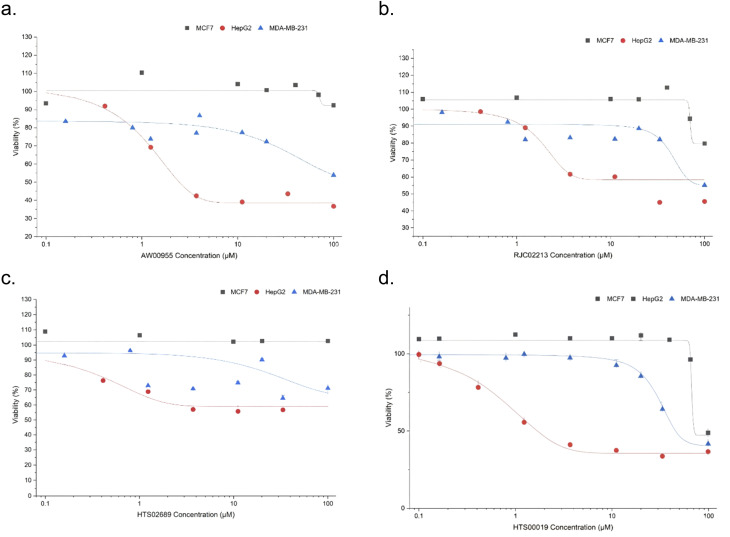
Concentration–response curves for cytotoxicity screening. Cell viability was determined by MTT assay^5^ after 48 hours of compound exposure against MDA-MB-231, MCF-7, and HepG2 cell lines. (a) AW00955, (b) RJC02213, (c) HTS02689, and (d) HTS00019. Data are presented as mean ± SD (*n* = 3). Note the exceptional potency of HTS00019 against HepG2 cells (IC_50_ = 1. 7 ± 0.22 µM) compared to the other tested compounds.

The selectivity profile assures additional mechanistic research. HepG2 cells, derived from a well-differentiated hepatoblastoma, express many characteristics of normal hepatocytes, such as the expression of drug metabolism enzymes and transporters. The high sensitivity of HepG2 cells to HTS00019 may result from the expression of a tissue-relevant molecular target or from dependence on a pathway that is particularly important for HepG2 cell survival (Table 1 and Fig. 2).^22^ Additionally, studies showed that tryptanthrin targets GSTP1 to induce senescence and increase susceptibility to apoptosis in liver cancer cells, providing precedent for GSTP1 as a viable target in HCC therapeutics.^23^

**Table 1:** Cytotoxicity of tested compounds against human cancer cell lines^a^

Compound	HepG2 IC_50_ (µM)	MDA-MB-231 IC_50_ (µM)	MCF-7 IC_50_ (µM)
HTS00019	1. 7 ± 0.22	86.29 ± 1.50	94.03 ± 1.80
AW00955	2.1 ± 0.05	>100	>100
RJC02213	8 ± 1.45	>100	>100
HTS02689	>100	>100	>100

a IC_50_ values represent mean ± SD from three independent experiments. ND: not determined.

### 3.2 Computational target deconvolution reveals GSTP1 and TRAP1 as primary molecular targets

The identification of molecular targets for the noteworthy cytotoxicity and selectivity of HTS00019 was a crucial part of this research. To accomplish this, we have utilized the Similarity Ensemble Approach (SEA) search server to carry out predictions for possible protein targets based upon the compound's structural properties. This chemoinformatics tool, which was validated for use in the identification of targets, operates under the assumption that structurally similar ligands have a tendency to act upon similar protein targets and thus perform ensemble similarity calculations.

From the results of the SEA analysis, there are several statistically significant target predictions, as shown in Table 2, and the members of the glutathione S-transferase family are highly represented in the top-ranked target predictions. The highest-ranked target prediction was GSTM2, with an *E*-value of 4.085 × 10^−44^ and a *Z*-score of 77.45, and the second highest-ranked target prediction was GSTP1, with an *E*-value of 2.146 × 10^−26^ and a *Z*-score of 45.63. The very low *E*-values suggest that there was a highly significant association between the structural features of HTS00019 and the well-characterized ligand space of the members of the GST family of enzymes. Other potential target proteins, such as phosphodiesterase 7B, COP9 signalosome complex subunit 5, ubiquitin carboxyl-terminal hydrolase 47, and TRAP1, also emerged from the SEA analysis, and each of these target proteins has an *E*-values below the 1.0 × 10^−5^ threshold.

**Table 2:** SEA search server target prediction results for HTS00019^a^

Target	Description	*E*-value	Max *T*_c_	Cut sum	*Z*-score
GSTM2	Glutathione S-transferase Mu 2	4.085 × 10^−44^	0.3377	3.7302	77.45
GSTP1	Glutathione S-transferase P1	2.146 × 10^−26^	0.3377	3.7302	45.63
PDE7B	cAMP-specific phosphodiesterase 7B	1.11 × 10^−16^	0.3824	1.0569	28.07
COPS5	COP9 signalosome complex subunit 5	1.088 × 10^−9^	0.3253	0.3253	15.64
USP47	Ubiquitin carboxyl-terminal hydrolase 47	7.608 × 10^−8^	0.2911	0.2911	12.33
TRAP1	Heat shock protein 75 kDa, mitochondrial	2.426 × 10^−7^	0.3133	0.5937	11.43

a Results filtered by organism (*Homo sapiens*) and relevance to cancer. Max *T*_c_: maximum Tanimoto coefficient. Targets with *E*-value < 1.0 × 10^−5^ are considered statistically significant.

The significance of the GST family in the SEA prediction scheme merits consideration in the context of the HepG2 selectivity. GSTP1 overexpression has been reported in various types of cancer, including notably hepatocellular carcinoma, in which it plays a role in the development and chemoresistance of the cancer.^5^

Moreover, studies linked the role of HSP90 and TRAP1 in HCC treatment, establishing TRAP1 as a validated target in this malignancy.^6^ The convergence of SEA predictions toward these two target classes: GSTP1 and TRAP1, provides a compelling mechanistic hypothesis for the observed phenotypic selectivity.

### 3.3 Reverse docking validates GSTP1 and TRAP1 as high-affinity targets

To validate and quantitatively evaluate the predictions generated by the SEA tool in an independent manner, a set of cancer-related protein targets were selected, and reverse docking analysis against these protein targets was performed using AutoDock Vina. The results obtained in the reverse docking study, as shown in Table 3, suggest HTS00019 as a high-affinity ligand, a hypothesis further tested through MD simulations to account for protein flexibility and solvent effects. For instance, significant levels of interaction are shown with PDE7A, TRAP1, and GSTP1, where Δ*G* = −9.9 kcal mol^−1^, Δ*G* = −9.8 kcal mol^−1^, and Δ*G* = −9.3 kcal mol^−1^, respectively. The high degree of agreement between the predictions obtained from the SEA method and the results obtained in the reverse docking study provides strong convergent evidence for the identification of GSTP1 and TRAP1 as major molecular targets. The hierarchy in the levels of binding affinity obtained in the docking study correlates well with the statistical significance ranking obtained in the SEA method, where the GST family and TRAP1 consistently appear in the top ranking positions as major molecular targets. Although PDE7A showed the highest docking affinity (−9.9 kcal mol^−1^), GSTP1 and TRAP1 were prioritized for further investigation due to their unique overexpression profiles in HCC and their documented roles in the development of drug resistance in HepG2 cells. This multi-target approach provides a more robust explanation for the observed 50-fold selectivity than a single-target inhibition of PDE7A.

**Table 3:** Reverse docking results: binding free energies of HTS00019 against predicted targets^a^

Rank	Target	PDB code	Description	Binding energy (kcal mol^−1^)
1	PDE7A	4Y2B	cAMP-specific phosphodiesterase 7A	−9.9
2	TRAP1	7C7B	TNF receptor-associated protein 1	−9.8
3	GSTP1	10GS	Glutathione S-transferase P1	−9.3
4	GSTM1	3GUR	Glutathione S-transferase Mu 1	−7.7
5	GSTA1	7BIB	Glutathione S-transferase A1	−7.6
6	USP47	8ITP	Ubiquitin carboxyl-terminal hydrolase 47	−7.2
7	COPS5	5M5Q	COP9 signalosome complex subunit 5	−7.1

a Binding free energy (Δ*G*) values were calculated using AutoDock Vina. RMSD values for re-docking validation were all <2.0 Å.

One interesting observation was that the compound showed higher binding affinity to GSTP1 in comparison to other GST isozymes. The compound showed binding affinities of −7.7 kcal mol^−1^ to GSTM1 and −7.6 kcal mol^−1^ to GSTA1, while binding to GSTP1 was 1.6 to 1.7 kcal mol^−1^ more favorable. Thus, the compound showed considerable isozyme selectivity in binding, as predicted by the computational model, and this observation correlates with the compound's cellular selectivity profile as well, as GSTP1 is the most abundant GST isozyme that is overexpressed in hepatocellular carcinoma.

### 3.4 Molecular basis of GSTP1 recognition: binding mode analysis

**Fig. 3:**
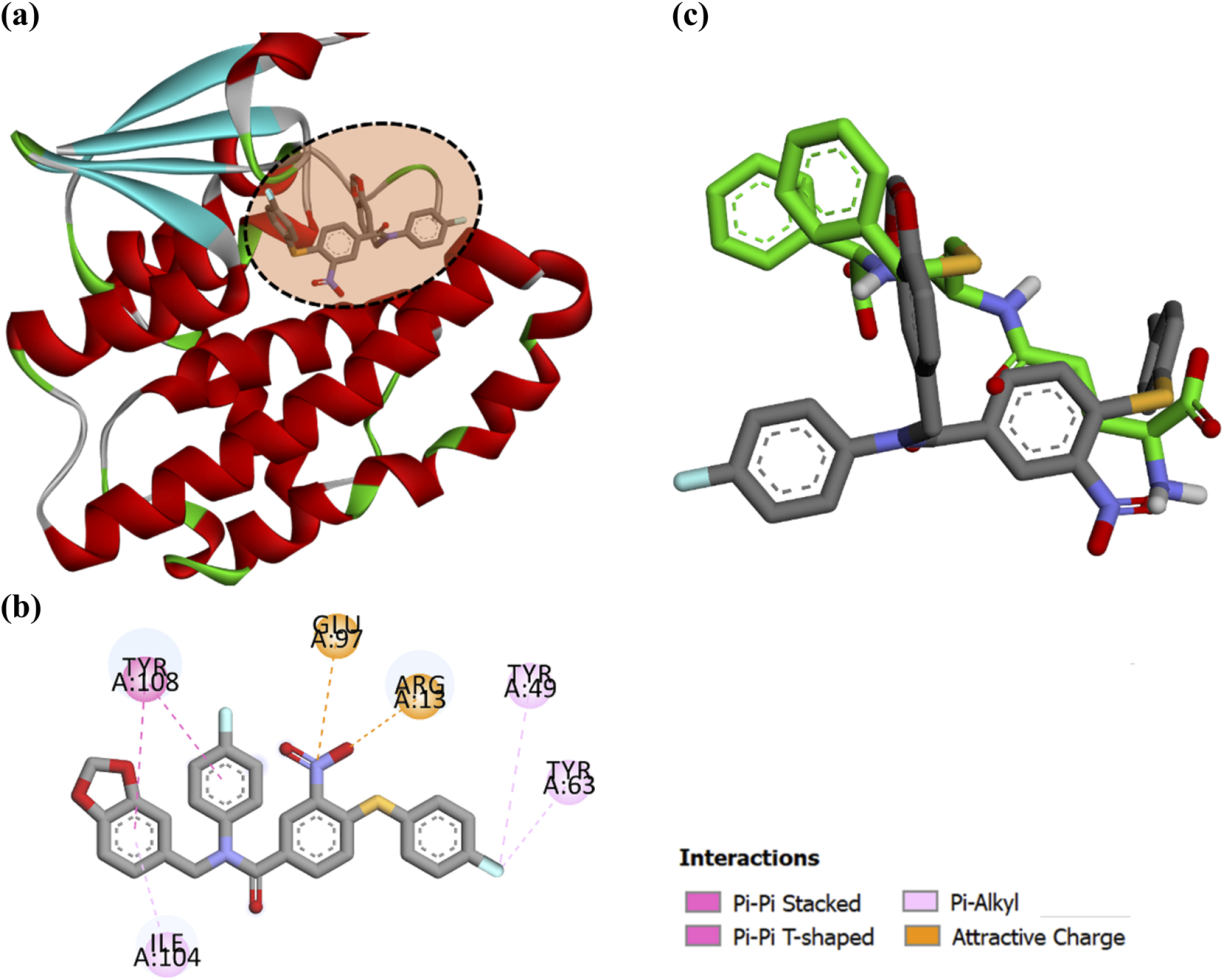
Predicted binding mode of HTS00019 in the GSTP1 active site. (a) Overall view of HTS00019 (shown as sticks with carbon atoms in gray) bound within the H-site of GSTP1 (PDB: 10GS), shown in solid ribbon representation. (b) Detailed view of key protein–ligand interactions. (c) Superposition of HTS00019 (gray) with the co-crystallized compound (TER117) (green) showing distinct binding orientations.

An in-depth examination of the docking-predicted binding mode of compound HTS00019 in the GSTP1 active site reveals mechanistic insight into the molecular basis of target recognition and selectivity. GSTP1 has two adjacent domains, G-site (glutathione binding site) and an all-helical C-terminal domain (H-site, substrate specificity determinant). The top-ranked docking pose of compound HTS00019 places the compound in the hydrophobic pocket of the H-site, consistent with the binding mode of GST inhibitors that compete with the binding of electrophilic substrates (as shown in Fig. 3).

The binding mode analysis (Table 4 and Fig. 3b) reveals a complex interplay of interactions, which all contributes to the stability of the complex of HTS00019 and GSTP1. Notably, a strong ionic interaction is anticipated between the positively charged Arg13 and HTS00019, which is located at a distance of 3.0 Å. This arginine residue, located at the entrance of the G-site, is known to be crucial in the stabilization of the glutathione backbone in the native complex of the enzyme and the substrate. It has also been shown to be involved in the binding of various GST inhibitors.^24^

**Table 4:** Predicted key interactions between HTS00019 and GSTP1 active site residues^a^

Residue	Interaction type	Distance (Å)	Functional role
Tyr108	Aromatic	4.6	H-site wall stabilization
Tyr49	Hydrophobic	4.0	G-site entrance
Tyr63	Hydrophobic	4.2	G-site entrance
Arg13	Ionic	3.0	Electrostatic anchoring
Glu97	Ionic	4.1	Electrostatic anchoring
Ile104	Hydrophobic	4.0	Hydrophobic anchoring in H-pocket

a Interactions analyzed using Discovery Studio Visualizer based on the top-scoring docking pose.

The major binding force between HTS00019 and the H-site is hydrophobic interactions. The aromatic side chain of Tyr108, which forms one face of the hydrophobic pocket within the H-site, is involved in π–π stacking interactions with the aromatic ring of HTS00019 at a distance of 4.6 Å. This interaction is particularly interesting because this residue is a key determinant of substrate specificity in GSTP1, and it has been shown to influence substrate specificity when mutated in this enzyme. In addition, hydrophobic interactions with Tyr49 at a distance of 4.0 Å and Tyr63 at a distance of 4.2 Å, both at the entrance of the G-site, could play a role in the binding stability and in the ability of this compound to interfere with glutathione binding.

The hydrophobic residue Ile104, in H-site pocket, makes close contact (4.0 Å) with HTS00019, contributing to the burial of the compound within the hydrophobic cavity. This interaction is noteworthy because Ile104 has been identified as a key residue differentiating GSTP1 from other GST isozymes, potentially contributing to the observed computational selectivity. The ionic interaction with Glu97 (4.1 Å) provides additional electrostatic stabilization and contribute to the overall binding energy favorability.

### 3.5 TRAP1 recognition: dual targeting potential of HTS00019

Considering the high predicted affinity of HTS00019 to TRAP1 (−9.8 kcal mol^−1^), we have extended the exploration of the binding mode to this mitochondrial chaperone, which is highly expressed in hepatocellular carcinoma.^6^

**Fig. 4:**
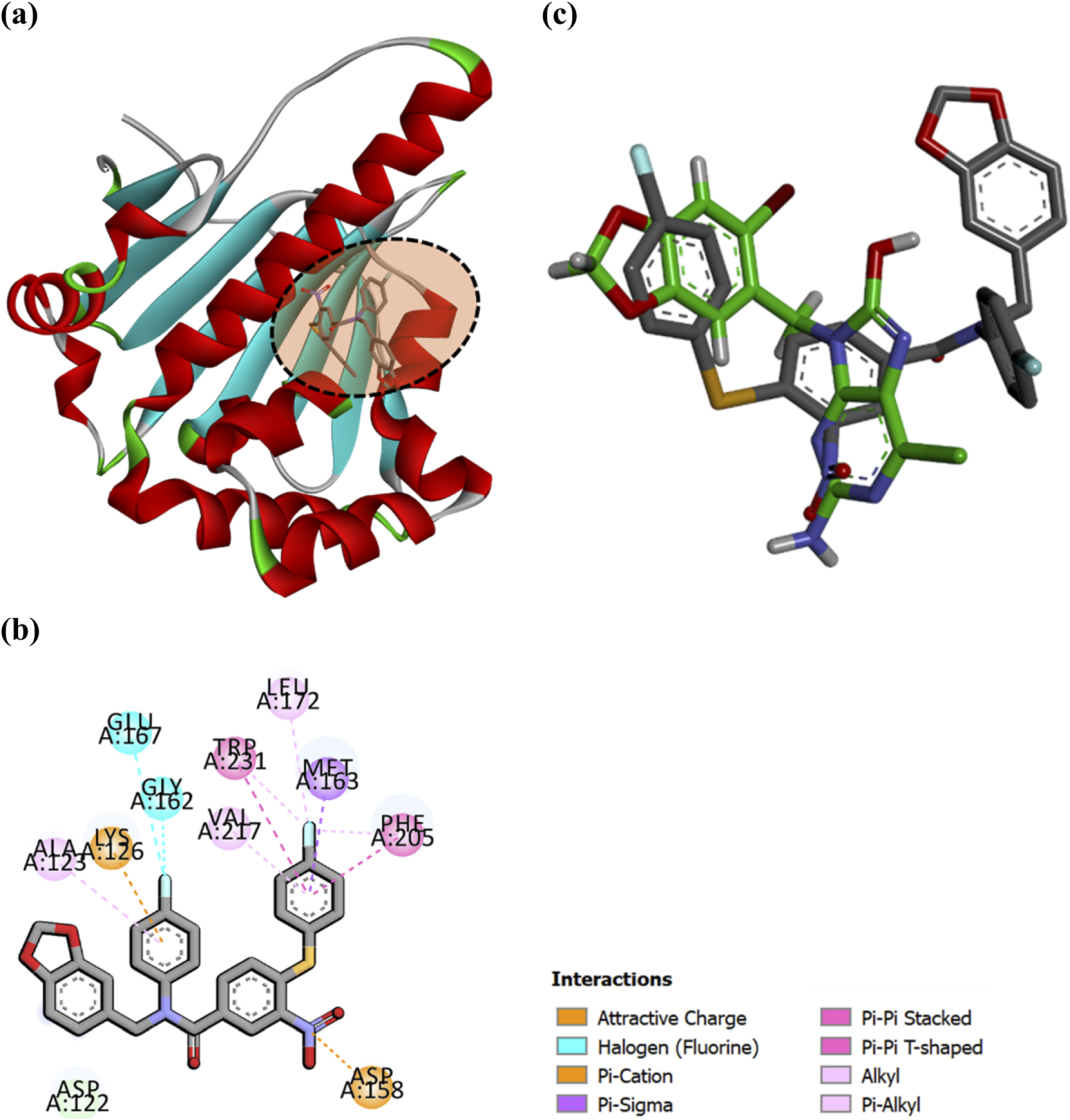
Predicted binding mode of HTS00019 in the TRAP1 active site. (a) Overall view of HTS00019 (shown as sticks with carbon atoms in gray) bound within the active site of TRAP1 (PDB: 7C7B), shown in solid ribbon representation. (b) Detailed view of key protein–ligand interactions. (c) Superposition of HTS00019 (gray) with the co-crystallized compound (SJT009) (green) showing distinct binding orientations.

The binding mode analysis of HTS00019 within the TRAP1 (Fig. 4 and Table 5) showed a diverse interaction profile. An interesting interaction performed within TRAP1 binding site is the formation of halogen bonds between halogen atom (Flour) on HTS00019 and backbone carbonyl oxygen atoms of Gly162 (2.8 Å) and Glu167 (3.5 Å). Halogen bonds, which involve the interaction between an electron-deficient halogen atom and a Lewis base, have emerged as valuable interactions in structure-based drug design due to their directionality and strength comparable to hydrogen bonds.

**Table 5:** Predicted key interactions between HTS00019 and TRAP1 active site residues^a^

Residue	Interaction type	Distance (Å)	Functional significance
Gly162	Halogen bond	2.8	Strong directional interaction
Glu167	Halogen bond	3.5	Strong directional interaction
Lys126	Pi-cation	4.0	ATP-binding site interaction
Met163	Hydrophobic	3.6	Pocket stabilization
Phe205	Aromatic	3.6	π–π stacking
Leu172	Hydrophobic	4.0	Pocket stabilization

a Interactions analyzed using Discovery Studio Visualizer based on the top-scoring docking pose.

A predicted π-cation interaction was recorded between the amino acid residue Lys126 and the aromatic ring system of the compound HTS00019 at a distance of 4.0 Å. This particular interaction is of significant interest because the amino acid residue Lys126 is highly conserved across all HSP90 family proteins and is of critical importance for the process of ATP binding and hydrolysis. The involvement of this particular residue suggests that the compound HTS00019 has the potential to act as an effective competitor for the TRAP1 binding site and the ATP molecule. Other hydrophobic interactions were noted with amino acid residues of the ATP-binding pocket of the protein, including Ala123 at 5.1 Å, Trp231 at 5.1 Å, Leu172 at 4.0 Å, Val217 at 5.1 Å, and Met163 at 3.6 Å.

The aromatic interaction with Phe205 (3.6 Å) provides additional π–π stacking stabilization. Phe205 is in a region of TRAP1 that undergoes conformational changes during the ATPase cycle, in which may influence the enzyme's conformational dynamics. The collective interactions observed in the TRAP1 binding mode suggest that HTS00019 has the potential to act as an effective TRAP1 inhibitor, which, combined with its GSTP1 inhibitory activity, positions it as a promising dual-target therapeutic candidate for hepatocellular carcinoma.

**Fig. 5:**
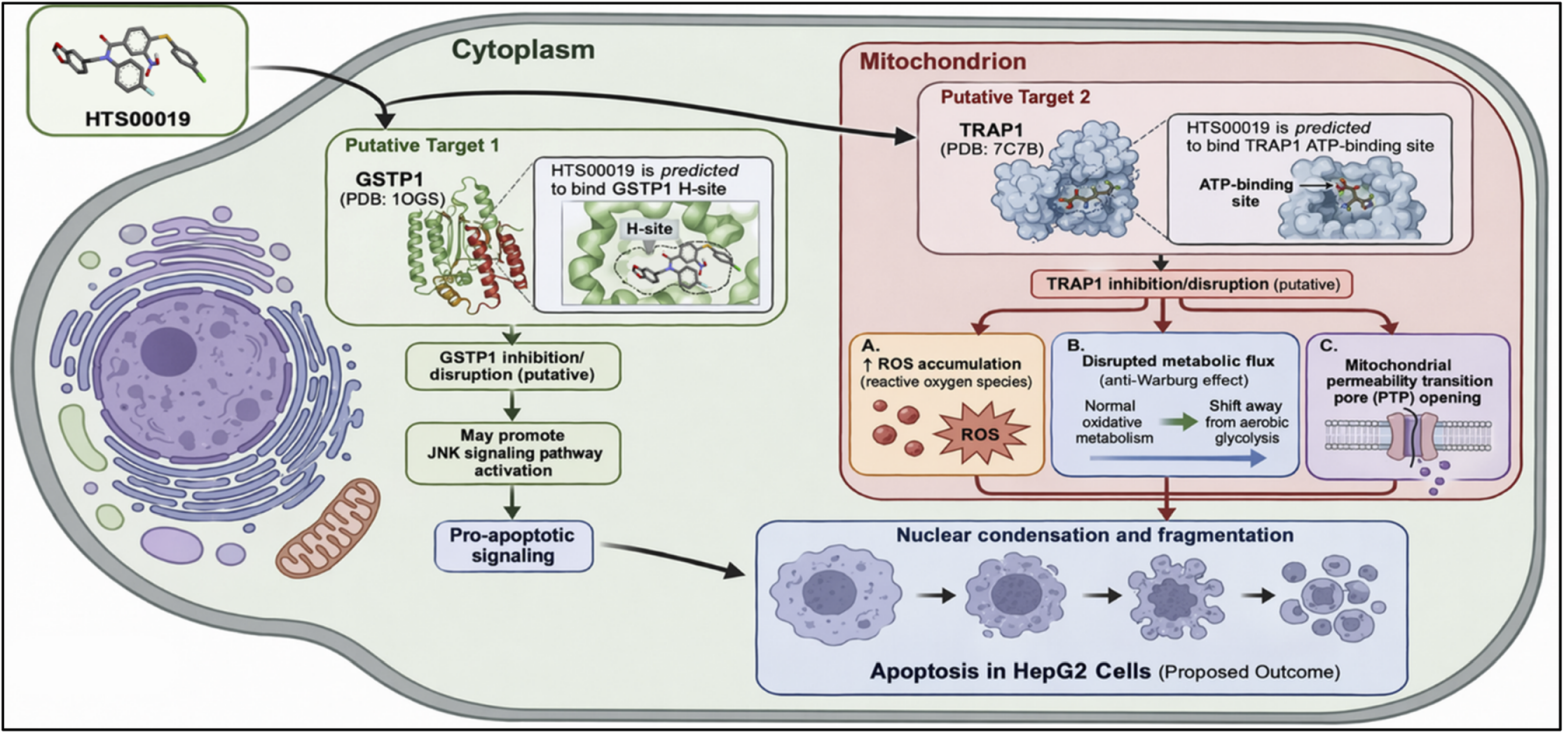
Mechanism of action of HTS00019 in HepG2 cells. The dual mechanism of cytotoxicity is exerted through two cellular compartments. The compound targets the cytoplasm where GSTP1 is inhibited, resulting in the activation of JNK. Simultaneously, the molecule localizes within mitochondria and inhibits TRAP1 leading to oxidative stress (ROS generation) and mitochondrial permeability transition. Both actions together lead to the induction of apoptosis.

This combined effect on cell disruption through the joint inhibition of GSTP1 and TRAP1 is illustrated in the scheme shown in Fig. 5.

### 3.6 Molecular dynamic stability and binding free energy (MM-GBSA) analysis

In order to determine the binding stability between HTS00019 and GSTP1 & TRAP1, a 100 ns molecular dynamics simulation was carried out. Control simulations were done with the help of co-crystallized compounds in complex with their respective proteins, *i.e.*, TER117 bound with GSTP1 & SJT009 with TRAP1, for comparison purposes. The stability of ligand–protein complex was determined in all cases by studying certain properties of protein such as RMSD, RMSF, ligand–protein interactions, and binding energy through MM-GBSA.

**Fig. 6:**
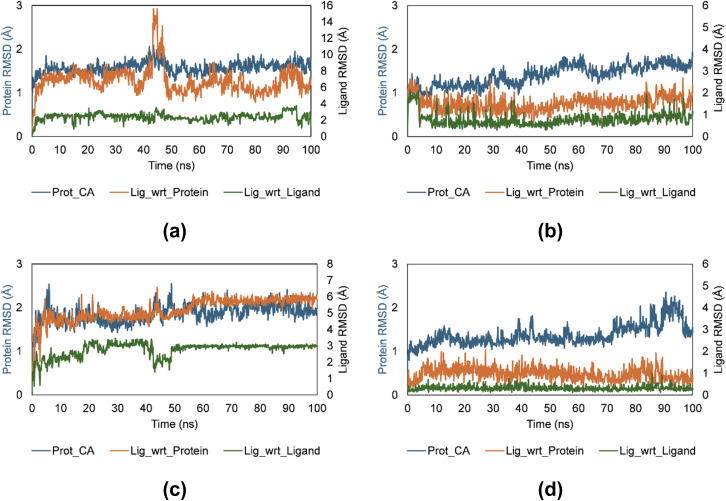
RMSD analysis of the four simulated systems; (a) HTS00019-GSTP1, (b) TER117-GSTP1, (c) HTS00019-TRAP1, and (d) SJT009-TRAP1. The RMSD of protein α-carbons (Prot_CA) is shown as a blue line, the ligand RMSD relative to the protein (Lig_wrt_Protein) as an orange line, and the internal ligand RMSD (Lig_wrt_Ligand) as a green line.

From RMSD calculations (Fig. 6), it was observed that there were differential stabilities associated with the simulated complexes. The complex formed by HTS00019 and GSTP1 was characterized by fast equilibrium, reaching equilibrium at 2.0 ns with a *C*_α_ RMSD value between 1.001 and 2.078 Å. On the other hand, the stability of the reference ligand TER117 in complex with GSTP1 is also similar, where RMSD value ranges from 0.902 to 1.934 Å. Nevertheless, the ligand-based RMSD analysis demonstrated that HTS00019 had a significant fluctuation inside the pocket with a range of 1.295–8.860 Å and even had a deviation of 15.524 Å after 45 ns, suggesting temporary instability or dislocation of the ligand. Meanwhile, the ligand TER117 bound much tighter to the protein, and its RMSD was between 0.610 and 2.681 Å. From internal RMSD calculations, HTS00019 remained structurally stable (0.896–3.757 Å), although still more flexible than TER117, which exhibited a lower internal deviation (0.288–2.146 Å).

For the TRAP1, its complex with HTS00019 also expressed early stabilization, reaching equilibrium at approximately 4.8 ns with RMSD values between 1.108 and 2.545 Å. The control complex with SJT009 showed comparable backbone stability (0.803–2.353 Å). Notably, HTS00019 displayed better binding stability within the TRAP1 pocket compared to GSTP1, with ligand RMSD values ranging from 3.926 to 6.562 Å and no extreme deviations observed. Nevertheless, SJT009 maintained tighter binding overall (0.291–2.071 Å). Internal RMSD analysis further confirmed ligand stability, with HTS00019 ranging from 1.394 to 3.457 Å, while SJT009 exhibited minimal structural deviation (0.109–0.980 Å).

**Fig. 7:**
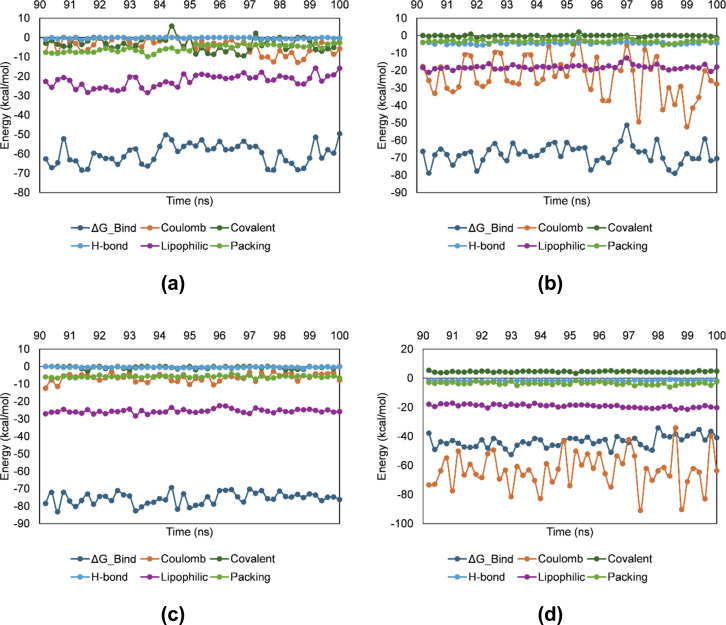
Dynamic changes in MM-GBSA components over the last 10 ns of the simulations time; (a) HTS00019-GSTP1, (b) TER117-GSTP1, (c) HTS00019-TRAP1, and (d) SJT009-TRAP1.

As can be seen from the analysis of the binding energies (Fig. 7 and Table 6), HTS00019 has the highest affinity towards TRAP1, with an average binding energy of −75.493 kcal mol^−1^, far higher than the value obtained for the reference compound SJT009, which was −43.488 kcal mol^−1^. On the contrary, HTS00019 had a lower affinity towards GSTP1 compared to TER117 (−59.981 *vs.* −67.917 kcal mol^−1^).

**Table 6:** Mean MM-GBSA energy contributions computed from the last 10 ns of molecular dynamics simulations for the HTS00019-GSTP1, TER117-GSTP1, HTS00019-TRAP1, and SJT009-TRAP1 complexes

Protein	Ligand	Δ*G*_bind_ (kcal mol^−1^)	Coulomb (kcal mol^−1^)	Covalent (kcal mol^−1^)	H-bond (kcal mol^−1^)	Lipophilic (kcal mol^−1^)	Packing (kcal mol^−1^)
GSTP1	HTS00019	−59.981 ± 5.230	−5.068 ± 3.391	−3.841 ± 3.147	−0.321 ± 0.315	−22.351 ± 3.160	−5.514 ± 1.826
	TER117	−67.917 ± 5.455	−23.715 ± 11.384	−0.573 ± 0.864	−4.363 ± 0.612	−18.128 ± 1.321	−3.211 ± 0.883
TRAP1	HTS00019	−75.493 ± 3.395	−6.355 ± 2.211	−0.309 ± 0.992	−0.461 ± 0.213	−25.539 ± 1.156	−5.802 ± 0.588
	SJT009	−43.488 ± 3.913	−63.443 ± 12.019	4.345 ± 0.136	−1.687 ± 0.243	−19.160 ± 1.089	−3.712 ± 0.878

Decomposition of the interaction energies indicated that coulombic contributions were most favorable for the reference complexes, with TER117-GSTP1 and SJT009-TRAP1 showing values of −23.715 and −63.443 kcal mol^−1^, respectively, compared to the weaker electrostatic contributions of HTS00019 (−5.068 kcal mol^−1^ for GSTP1 and −6.355 kcal mol^−1^ for TRAP1). Conversely, HTS00019 exhibited the strongest lipophilic interactions, with energies of −22.351 and −25.539 kcal mol^−1^ in complex with GSTP1 and TRAP1, respectively, exceeding those of TER117 (−18.128 kcal mol^−1^) and SJT009 (−19.160 kcal mol^−1^).

In terms of solvation effects, HTS00019 showed more favorable solvent polarization energies in both complexes (−5.514 and −5.802 kcal mol^−1^ for GSTP1 and TRAP1, respectively) compared to TER117 (−3.211 kcal mol^−1^) and SJT009 (−3.712 kcal mol^−1^), suggesting stronger solvent-mediated stabilization. In contrast, hydrogen bonding contributions were weaker for HTS00019 (−0.321 and −0.461 kcal mol^−1^ for GSTP1 and TRAP1, respectively) relative to TER117 (−4.363 kcal mol^−1^) and SJT009 (−1.687 kcal mol^−1^), indicating that its binding is less dependent on hydrogen bond formation.

**Fig. 8:**
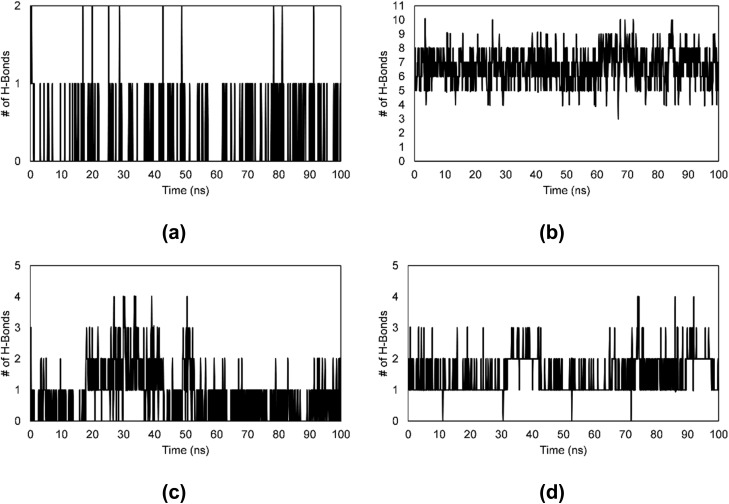
Frequency of the hydrogen bonds interactions over the 100 ns of (a) HTS00019-GSTP1, (b) TER117-GSTP1, (c) HTS00019-TRAP1, and (d) SJT009-TRAP1.

Following the stability analysis, a comparative evaluation of the interaction modes of the three compounds was performed. The number of hydrogen bonds was first examined (Fig. 8). All systems reached a dynamic equilibrium in hydrogen bond formation; however, notable differences were observed among the ligands. HTS00019 maintained approximately one hydrogen bond with minor fluctuations in both GSTP1 and TRAP1 complexes, whereas SJT009 stabilized at around two hydrogen bonds. In contrast, TER117 exhibited the highest degree of stability, consistently forming approximately seven hydrogen bonds throughout the simulation.

**Fig. 9:**
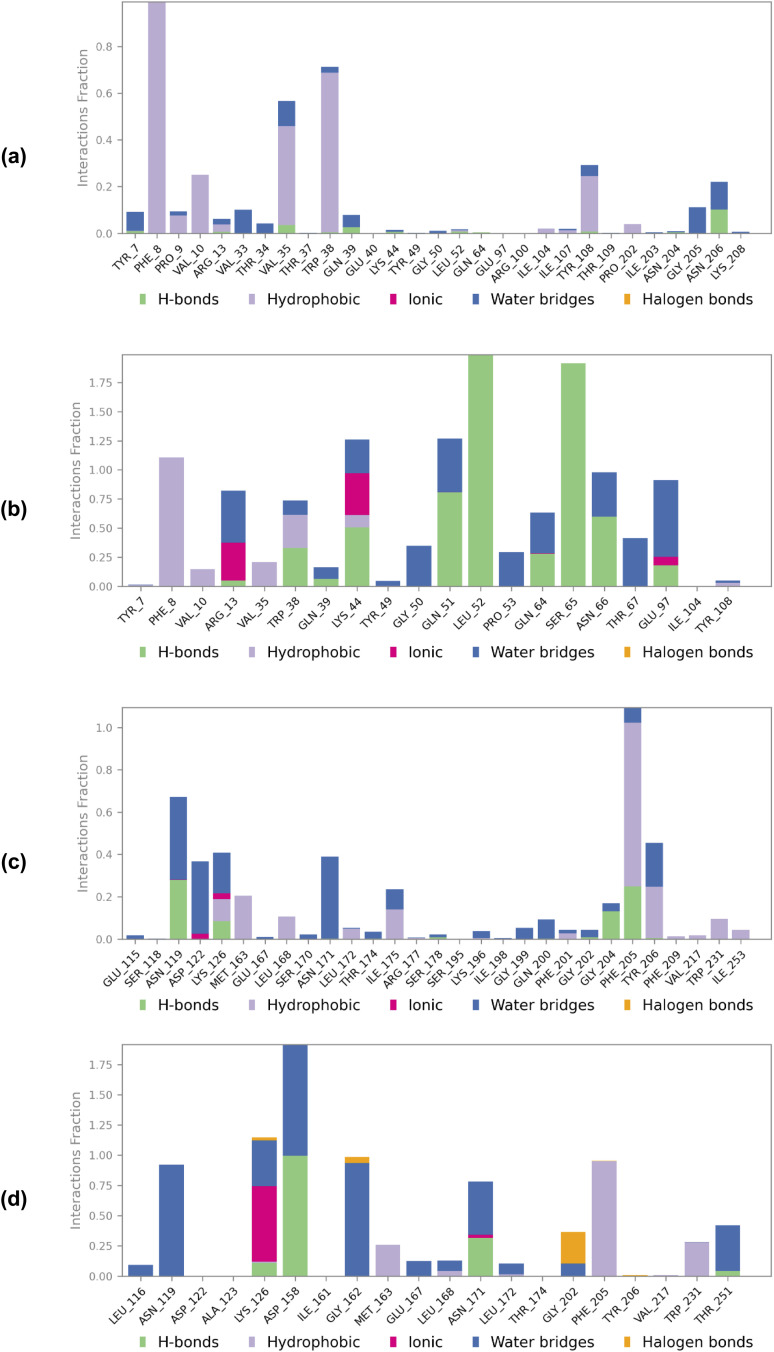
Interaction profiles of the simulated complexes over 100 ns, showing residue-level contacts for (a) HTS00019-GSTP1, (b) TER117-GSTP1, (c) HTS00019-TRAP1, and (d) SJT009-TRAP1.

The interaction patterns of HTS00019 with GSTP1 and TRAP1 residues were further analyzed over the course of the simulation (Fig. 9). In comparison to TER117, which remains tightly localized within the GSTP1 binding pocket in its co-crystal conformation, HTS00019 exhibited more spatially dispersed interaction sites. This behavior suggests increased conformational adaptability during binding, with interactions dominated primarily by hydrophobic contacts and water bridges. A similar trend was observed in the TRAP1 complex when compared to SJT009, where HTS00019 again displayed a broader distribution of interacting residues, indicative of dynamic binding behavior. However, in TRAP1, HTS00019 formed a more diverse interaction profile, involving not only water bridges and hydrophobic contacts but also a greater contribution from hydrogen bonding relative to its interaction pattern in GSTP1.

**Fig. 10:**
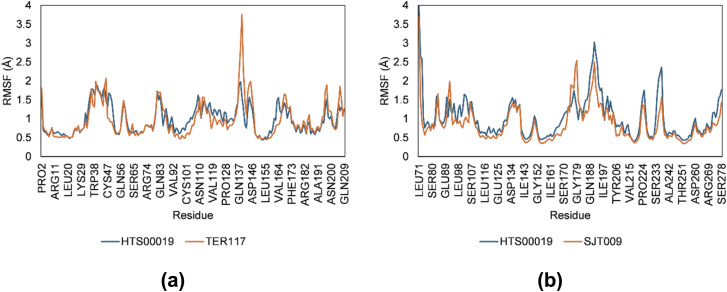
RMSF analysis of amino acid residues in (a) GSTP1 and (b) TRAP1 when bound to HTS00019 (blue line) and the reference ligands TER117 and SJT009 (orange line).

RMSF analysis further demonstrated that HTS00019 effectively preserves the dynamic behavior of key functional regions in both targets. In GSTP1, the compound influenced the principal active-site segments (Tyr7-Arg13, Trp38-Pro53, Gln64, Ser65, and Tyr108), while in TRAP1 it engaged critical binding-site residues (Ala123, Asp158-Asn171, Phe205, Trp231, and Thr251). Notably, the flexibility profiles of these regions remained highly consistent with those observed for the reference ligands TER117 and SJT009 (Fig. 10). This overlap in residue-level fluctuations indicates that HTS00019 does not induce additional structural perturbations, but rather maintains the intrinsic dynamic stability of the binding sites. Such behavior supports its ability to achieve stable accommodation within both binding pockets without compromising local structural integrity.

### 3.7 *In silico* toxicological profiling, structural mutagenicity assessment, and nitrosamine risk discussion

To address the toxicological liabilities of HTS00019, a combined structure-based and *in silico* toxicity assessment was performed. Two related but distinct concerns are addressed separately: (i) the intrinsic toxicological profile of the parent compound as predicted by ProTox-3.0,^25^ and (ii) the structural basis of nitrosamine and genotoxic impurity risk relevant to the compound's chemical architecture.

#### 3.7.1 Nitrosamine and nitroreduction risk assessment

Nitrosamine risk assessment is primarily a manufacturing and storage impurity-control concern governed by ICH M7 and ICH Q3C guidelines, wherein N-nitroso impurities may arise from nitrosating conditions involving amine precursors, contaminated reagents, solvents, or degradation pathways. A full regulatory nitrosamine risk assessment therefore requires complete knowledge of the synthetic route, reagents, excipients, formulation, and storage conditions, supported by confirmatory analytical testing such as GC-MS/MS or LC-HRMS. Since HTS00019 was obtained as a commercially sourced screening compound (Maybridge/Thermo Fisher Scientific), no in-house synthesis data are available and a complete route-based regulatory nitrosamine assessment cannot be provided. The current evaluation is accordingly a preliminary structure-based hazard discussion, not a definitive regulatory assessment.

From a structural standpoint, HTS00019 contains a nitro aromatic functional group (–NO_2_), which is a recognized structural alert in the context of ICH M7 mutagenic impurity classification. Aromatic nitro compounds are subject to enzymatic nitroreduction by bacterial and mammalian nitroreductases, generating nitroso and hydroxylamine intermediates capable of direct DNA adduct formation. This nitroreduction pathway is the primary mechanistic basis for Ames test positivity in nitroaromatic compounds. Although this structural alert pertains to the parent compound itself rather than to a co-formed nitrosamine impurity, it is directly relevant to the observed mutagenicity prediction and constitutes a legitimate ICH M7 cohort-of-concern flagging scenario. No secondary amine functionality is evident in the structure of HTS00019 that would independently serve as a classical nitrosamine precursor amine; however, the presence of the nitro group itself generates a genotoxicity signal that subsumes the nitrosamine concern within the broader mutagenicity framework.

#### 3.7.2 ProTox-3.0 toxicological profile

As shown in Table 7, the ProTox-3.0 platform^25^ predicted an oral LD_50_ of 1500 mg kg^−1^, placing HTS00019 in toxicity class 4 (harmful if swallowed). Importantly, this acute systemic toxicity classification reflects a single endpoint and must not be interpreted as an overall safety assessment. ProTox-3.0 employs independent QSAR/machine-learning models trained on curated toxicology databases (including Tox21, ToxCast, and ChemBL) for each endpoint, and each prediction is expressed as a probability of activity. Table 7 summarizes the full predicted toxicological profile.

**Table 7:** Predicted toxicological profile of HTS00019*via* ProTox-3.0

Category	Parameter	Result	Probability
Acute tox	Predicted LD_50_	1500 mg kg^−1^	67.38% (accuracy)
Organ tox	Hepatotoxicity	Active	0.51
End points	Mutagenicity	Active	0.87
	Immunotoxicity	Active	0.99
	Carcinogenicity	Active	0.60
	Cytotoxicity	Inactive	0.59
Stress pathways	p53 (DNA damage)	Inactive	0.74
	ATAD5 (genotoxicity)	Inactive	0.92
Off-targets	16 toxicity targets	No binding	

HTS00019 was predicted as active for mutagenicity (probability 0.87), immunotoxicity (0.99), carcinogenicity (0.60), and hepatotoxicity (0.51). These are not experimental findings; they represent computational hazard signals derived from structural similarity to known toxic chemical space. Nonetheless, a mutagenicity probability of 0.87 represents a high-confidence prediction that warrants serious consideration at the hit evaluation stage, not dismissal.

The mutagenicity alert is mechanistically attributable to the nitroaromatic scaffold of HTS00019. Nitroaromatic compounds are among the most robustly flagged structural alerts for Ames test positivity in all major QSAR mutagenicity platforms, including Derek Nexus, SARAH, and VEGA, and this finding is entirely consistent across *in silico* models. The predicted mutagenicity is therefore a structurally rationalized prediction, not an artifact. It is critical to distinguish this from the p53 and ATAD5 stress-pathway predictions, both of which were inactive (probabilities 0.74 and 0.92, respectively). These endpoints model cell-based transcriptional stress responses activated by broader genotoxic insults in mammalian systems, whereas the Ames mutagenicity endpoint captures direct bacterial DNA base-pair and frameshift mutation events mediated by reactive metabolites. The inactive p53/ATAD5 predictions thus indicate that activation of these specific mammalian stress response pathways was not predicted—they do not contradict or override the mutagenicity alert, which operates through a mechanistically distinct detection system. The genotoxicity profile of HTS00019 therefore remains unresolved and requires experimental resolution through the Ames test, the *in vitro* micronucleus assay, and/or the chromosomal aberration assay.

The carcinogenicity prediction (probability 0.60) shares a plausible mechanistic basis with the mutagenicity alert: genotoxic carcinogenicity driven by DNA adduct-forming intermediates is the expected downstream consequence of nitroreduction activity and is a well-established mechanistic link in the nitroaromatic pharmacotoxicology literature. The immunotoxicity prediction (probability 0.99) is the highest-confidence alert in the profile and may reflect structural features shared with compounds known to perturb immune cell function, though the precise structural basis for this prediction cannot be assigned without further fragment analysis. Both findings indicate that HTS00019 should not be described as a preclinically safe candidate at this stage.

The hepatotoxicity prediction (probability 0.51) is borderline and reflects a low-confidence signal. However, given that the compound is under investigation in a hepatocellular context, it warrants explicit attention. The selective cytotoxicity of HTS00019 toward HepG2 cells relative to MCF-7 and MDA-MB-231 lines is an activity-based observation from a cancer cell line and should not be extrapolated to hepatocyte safety. HepG2 cells are derived from a hepatoblastoma with a transformed metabolic phenotype that differs substantially from normal hepatocytes. Cytotoxicity testing against non-malignant hepatic cell models such as LO2 or HepaRG cells is required to define a meaningful therapeutic index and to determine whether the hepatotoxicity prediction reflects cancer-cell-selective toxicity or a genuine hepatic safety liability.

#### 3.7.3 Metabolic and off-target profile

Analysis of 16 major toxicological target receptors showed no predicted binding, suggesting a low probability of systemic receptor-mediated off-target effects. CYP450 profiling indicated metabolic inactivity across CYP1A2, 2C19, 2D6, 3A4, and 2E1, with the exception of CYP2C9 inhibition. This metabolic profile reduces the anticipated risk of reactive metabolite generation from major oxidative pathways, though the CYP2C9 interaction should be noted for potential drug–drug interaction screening in future studies.

In summary, the *in silico* toxicological profile indicates that HTS00019 is an early-stage phenotypic hit with promising HepG2-selective activity but with important and structurally rationalized toxicological liabilities. The nitroaromatic functionality represents the principal structural concern: it is a recognized ICH M7 cohort-of-concern alerting feature, a mechanistically plausible basis for the high-confidence mutagenicity prediction, and the most likely driver of the carcinogenicity signal. Further development should include: (1) experimental mutagenicity assessment (Ames test, *in vitro* micronucleus assay); (2) cytotoxicity profiling against non-cancerous hepatic cell lines to establish a therapeutic window; (3) CYP2C9 inhibition characterization; and (4) targeted analytical impurity screening if the compound is advanced beyond the phenotypic screening stage. Medicinal chemistry optimization should prioritize removal or bioisosteric replacement of the nitroaromatic moiety as the primary lead optimization objective, with the goal of eliminating the mutagenicity alert while retaining the dual GSTP1/TRAP1 pharmacophoric features responsible for HepG2-selective activity.

However, HTS00019 was classified as being Inactive when it comes to both p53 (0.74) and ATAD5 (0.92) stress-response pathways. It is especially noteworthy that the absence of ATAD5 activity, which is an incredibly reliable marker of genotoxic DNA damage, suggests that HTS00019 is not capable of causing either chromosomal instability or any other DNA-related lesions.

On the contrary, the prediction related to hepatotoxicity was marked as Active (0.51). However, taking into account the function of HTS00019 as a highly effective HCC inhibitor, which selectively affects liver cancer cells (HepG2, IC_50_ = 1. 7 µM), one may conclude that such a classification is related to the higher affinity to liver-derived cells but not toxicity to hepatocytes themselves. This is further corroborated by the “Inactive” prediction for general cytotoxicity (0.59), reinforcing the phenotypic screening results that the compound's cell-killing effect is targeted and selective rather than broad and non-specific.

Moreover, analysis of 16 major toxicity targets (including receptors such as ESR1, PDE4D, and HRH1) showed no probable binding. This indicates a low probability of systemic off-target side effects. Metabolic profiling showed that HTS00019 is primarily “Inactive” against most Cytochrome P450 isoforms (CYP1A2, 2C19, 2D6, 3A4, 2E1), with the exception of CYP2C9. This metabolic stability reduces the risk of reactive metabolite formation.

## 4. Conclusions

In this study, HTS00019 was identified as a highly potent and selective phenotypic hit against HepG2 cells, with an IC_50_ value of 1. 7 ± 0.22 µM and marked selectivity over the two tested breast cancer cell lines. The integrated computational workflow used here refined target prioritization beyond static docking and supported GSTP1 and TRAP1 as the most plausible candidate targets associated with the observed cytotoxic profile. Docking and interaction analysis suggested favorable binding of HTS00019 within the GSTP1 H-site and within the ATP-associated binding region of TRAP1, while 100 ns molecular dynamics simulations confirmed stable accommodation of the compound in both proteins.

Importantly, the dynamic and energetic analyses did not support identical behavior across the two targets. HTS00019 showed greater positional fluctuation in GSTP1 than the co-crystallized reference ligand, whereas its TRAP1 complex remained comparatively more stable and exhibited the most favorable MM-GBSA binding free energy among the evaluated HTS00019 complexes. These findings strengthen the hypothesis that TRAP1 may contribute substantially to the observed cellular phenotype, while GSTP1 remains a biologically relevant and computationally supported candidate target.

Furthermore, *in silico* toxicological profiling revealed a dual picture that must be transparently communicated. While an estimated oral LD_50_ of 1500 mg kg^−1^ places HTS00019 in toxicity class 4, and metabolic profiling indicated stability against most CYP450 isoforms—reducing the anticipated risk of reactive metabolite generation from major oxidative pathways—several endpoint-specific hazard signals were identified that preclude a premature safety designation. HTS00019 was predicted as Active for mutagenicity (probability 0.87), immunotoxicity (0.99), carcinogenicity (0.60), and hepatotoxicity (0.51) by ProTox-3.0. The mutagenicity and carcinogenicity alerts are structurally rationalized by the nitroaromatic moiety of HTS00019, a recognized ICH M7 cohort-of-concern structural feature associated with nitroreduction-mediated DNA reactivity. Although p53 and ATAD5 stress-pathway predictions were inactive, these endpoints operate through mechanistically distinct mammalian stress-response systems and do not override the Ames-relevant mutagenicity signal. Accordingly, HTS00019 should be regarded as an early-stage hit with promising selective activity rather than a confirmed safe preclinical candidate, and further advancement requires experimental genotoxicity testing, normal hepatocyte safety profiling, and targeted structural optimization directed at eliminating the nitroaromatic alerting feature while preserving the dual GSTP1/TRAP1 pharmacophoric profile.

While these results provide a strong mechanistic hypothesis for the observed phenotypic activity, future work must focus on biochemical validation through purified enzyme assays and efficacy studies in HCC xenograft models. Ultimately, the dual-target profile of HTS00019 against GSTP1 and TRAP1 represents a robust strategy for overcoming chemoresistance and improving outcomes in hepatocellular carcinoma.

## Author contributions

Belal O. Al-Najjar: conceptualization, investigation, data curation, writing – original draft. M. Helal: methodology, validation, writing – review and editing. Fadi G. Saqallah: visualization, writing – review and editing. B. Bandy: supervision, writing – review and editing.

## Conflicts of interest

The authors declare no conflict of interest.

## Data Availability

All data supporting the findings of this study are available within the article.
